# Variation in antioxidant capacity, antioxidant activity and mineral composition during flower development of oil-bearing rose (*Rosa damascena* Mill.)

**DOI:** 10.1038/s41598-023-44461-4

**Published:** 2023-10-12

**Authors:** Damla Önder

**Affiliations:** Department of Biology, Faculty of Arts and Sciences, Süleyman Demirel University, Isparta, 32260 Turkey

**Keywords:** Biotechnology, Plant biotechnology

## Abstract

Oil-bearing rose is an economically important rose species with a wide range of uses such as cosmetics, perfumery, food and health, but the changes in antioxidant capacity and antioxidant activity during flower development are not well understood. The antioxidant content and free radical scavenging properties of oil-bearing rose flowers are used in the cosmetic industry to modulate skin damage, and in the food industry as a source of antioxidants and sweeteners. The present investigation was carried out to explore the antioxidant capacity, antioxidant enzyme activity, and the composition and distribution of minerals in petals of oil-bearing rose at five flower development stages. The total antioxidant capacity of petals was determined using CUPRAC, DPPH, FRAP, FIC and ABTS methods. The antioxidant capacity of petals decreased during the flower development, suggesting that flowers in stage I and II are valuable sources of antioxidants. CUPRAC, DPPH, FRAP, FIC and ABTS scavenging activity of the petals at various developmental stages are strongly and positively correlated with each other. The activity of the antioxidant enzymes; superoxide dismutase, catalase, glutathione reductase, ascorbate peroxidase was highest at the bud stage (stage I), whereas the fully opened flowers (stage V) exhibited the lowest activity in oil-bearing rose petals. During the development of flower, malondialdehyde (MDA) content increased significantly from stage I to stage III and decreased at stage IV. Here we detected the contents of 15 elements in petals, some of them, especially calcium, magnesium, potassium and phosphorus showed significant changes during rose flowering. Generally, the highest mineral content was observed in stage I while the lowest content observed in stage V of flower development. These results showed a close link between flower development, antioxidant capacity, enzymatic antioxidant activity and mineral content, with stage I exhibiting the best antioxidant activity, mineral content and free radical scavenging potential. This work will serve as a baseline for understanding the possible roles of antioxidant capacity, antioxidant enzymes, mineral content and their interactions in the regulation of flower development.

## Introduction

The oil-bearing rose (*Rosa damascena* Miller) is a hybrid between *R. gallica* and *R. phoenicia* and is an important member of Rosaceae family because its flowers are used in essential oil production. The flowering period of oil-bearing rose extends from late April to early June, and flower development from bud stage to full bloom takes approximately 11–13 days^1^. Oil-bearing rose flowers are collected by hand in the early hours (4:00–10:00 A.M.) of the morning and used for industrial purposes immediately after harvest. Approximately 55–60% of the flowers of the oil-bearing rose produced are used for rose oil and rose water production, 40–45% for rose concrete and rose absolute and 1–2% for food purposes^2^.

In traditional medicine, oil-bearing rose flowers have been used as for chest and abdominal pains, menstruation and digestive ailments (mild laxative for constipation), and as a cardio-tonic agent to strengthen the heart^3,4^. Rose flowers are used traditionally as food and medicinal purposes, not only for their visual appeal, sweet taste and flavor, but also for their high antioxidant capacity and antioxidant enzyme activity. There are numerous known sources of natural antioxidants, including edible flowers. Essential oil^5^, decoction^6^, absolute^7^, aqueous^8^, methanol^1^ and ethanol^5^ extracts of oil-bearing rose are used because of their antioxidant activity. Oil-bearing rose flowers possess one of the highest antioxidant activity and have been tested as a caffeine-free tea making source^9^. Therefore, the high antioxidant capacity and activity of oil-bearing rose flowers are used as a source of antioxidants and sweeteners in the food industry and for therapeutic purposes in traditional medicine, as well as their beneficial effects in scavenging free radicals^3^. Furthermore, the mineral compositions found in roses are among the alternative sources of nutrition that are vital to healthy functioning of an organism.

Since petals are flower organs that primarily determine the commercial importance of flowers, emphasis has been given to the cultural, physiological and biochemical processes that occur during flower development^10,11^. Flower development is a progressive oxidative degradation process and developmental processes have been shown to be significantly influenced by reactive oxygen species (ROS), which are produced by metabolic processes in organelles^12^. A synergistic antioxidant defensive system, which enzymes like catalase (CAT), peroxidase (POX), superoxide dismutase (SOD), and glutathione reductase (GR) has been theorized to protect cells from damage caused by ROS^13,14^. Antioxidant enzymes typically have the ability to delay or prevent the initiation and spread of oxidative chain reactions^15^. Additionally, these compounds function as metal chelators to inhibit the production of hydroxyl radicals^16^. Notably, enzymatic defense systems involving superoxide dismutase (SOD), catalase (CAT), glutathione reductase (GR) and ascorbate peroxidase (APX) also contribute to ROS scavenging along with antioxidant compounds. The production of ROS such as superoxide (O_2_^−^) and hydrogen peroxidase (H_2_O_2_) by cells is an indication of plants development^17,18^. SOD, CAT, GR and APX are important enzymes playing crucial roles in antioxidant defense. SOD catalyzes the dismutation of O_2_^−^ to O_2_ and H_2_O_2_, and CAT, APX and GR then catabolize H_2_O_2_ into H_2_O and O_2_, thereby limiting the additional radical production from H_2_O_2_^19,20^. H_2_O_2_ is a potent oxidizing agent that can activate the signaling pathway to stimulate cellular proliferation or differentiation^21^. It is produced in the biological system by antioxidant enzymes such as SOD^22^. The increase in H_2_O_2_ content causes the formation of hydroxyl radical (^**·**^OH), which initiates lipid peroxidation and damage to cellular components^23^. Therefore, the regulation of ROS production by enzymatic antioxidants is of great interest in biological research.

The ability of redox compounds in food and biological systems to scavenge free radicals is known as antioxidant capacity. The capacity of antioxidant enzymes have been reported to play an important role in the regulation of flower development^24^. However, there is no agreement on an ideal method for estimating the antioxidant capacity^25^. The cupric reducing antioxidant capacity **(**CUPRAC) assay uses copper (II)-neocuproine reagent as the oxidizing agent and is a very fast way to evaluate the antioxidant activity of extracts in a short time. In this method, the increase in absorbance was measured at 450 nm based on the reduction of copper(II)-neocuproin to the highly colored copper(I)-neocuproin chelate as a result of the color change from light blue to orange yellow. Over other chromogenic reagents (such as 2,2′-azino-bis-(3-ethylbenzothiazoline-6-sulfonic acid) (ABTS), 2,2-diphenyl-1-picrylhydrazyl (DPPH)), CUPRAC has the advantages of being more stable and accessible^26^. Using an assay for the reduction of Fe^3+^ to Fe^2+^, the ferric reducing antioxidant power (FRAP) technique determined the reducing capacity of petal samples. The Fe^3+^/ferricyanide complex is converted to the ferric form in the presence of reducing agents that also serve as antioxidants in the samples^27^. FRAP acts as a reducing agent with its ability to donate a single electron or hydrogen for the reduction of antioxidants^28^. FRAP capacity is due to the presence of antioxidant compounds involved in electron transfer. These antioxidant compounds have the ability to neutralize free radicals, convert them into stable compounds and terminate the reactions initiated by free radicals^29^. The most common method for assessing the radical-scavenging capacity of plant extracts is the DPPH assay^30^. The antioxidants are the parts of plants that can physically quench the stable purple DPPH radical, turning it into a yellow DPPH radical, which can be seen^21,31^. The DPPH test measures the radical scavenging ability of extracts. Iron causes the formation of ^**·**^OH by stimulating lipid peroxidation^32^. Iron ions are also considered the most effective pro-oxidants widely used in the food industry. The antioxidant capacity in ethanolic extracts of *R. damascena* showed a high antioxidant capacity when compared with the standard antioxidant *L*-ascorbic acid^33^. The antioxidant capacity of the fresh flower extract of *R. damascena* flowers was higher than the spent flower extract^34^. The antioxidant effect of oil-bearing rose and its inhibitory effect on lipid oxidation were evaluated in an in vivo study. The results suggested that oil-bearing rose showed a potent antioxidant and lipid peroxidation inhibitory effect comparable to tocopherol, and the rose could be considered as a medicinal source for the treatment and prevention of many free radical diseases^4^.

Since antioxidant compounds and antioxidant activity of enzymes are important part of flower development, it is necessary to investigate antioxidant content and antioxidant enzyme activities to better understand their changes during flower development to determine a suitable period for harvesting. Though, there are some reports on antioxidant capacity and antioxidant activity of rose petals^1,35,36^, to the best of our knowledge, there is no available information on antioxidant enzyme activity and antioxidant capacity at the different developmental stages of oil-bearing rose. Mineral distribution and metabolic changes in rose flowers that occur during the growth of oil-bearing rose petals are yet to be studied. The present study was conducted to characterize the mineral content, antioxidant capacity and enzymatic antioxidant activity at five distinct development stages of oil-bearing rose, which is traditionally used as cosmetic, pharmaceutical and food. Thus, flower development periods with high ratios of these components can be determined and flowers in this period can be preferentially harvested at a suitable development period.

## Results

### Total antioxidant capacity of oil-bearing rose petals at different flower development stages

**Figure 1:**
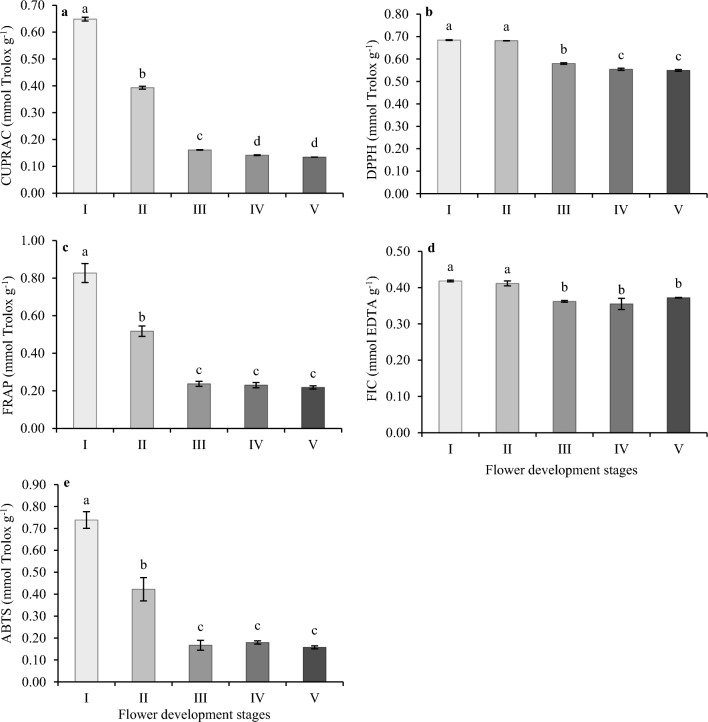
Total antioxidant capacities of oil-bearing rose petals during the flower development. (**a**) CUPRAC radical scavenging assay, (**b**) DPPH radical scavenging assay, (**c**) FRAP radical scavenging assay, (**d**) FIC radical scavenging assay, (**e**) ABTS radical scavenging assay. Data are represented as mean ± standard deviation (n = 3). Mean values with a different letters were significantly different (p ≤ 0.05).

Different in vitro antioxidant test methods could be used to determine the total antioxidant capacity (TAC), a crucial quality parameter for medicinal and edible plants. Each assay has its advantages and disadvantages, but not a single assay could measure antioxidant capacity accurately. Thus, it was necessary to determine antioxidant capacity of oil-bearing rose petals by CUPRAC, DPPH, FRAP, ferrous ion-chelating** (**FIC) and ABTS assays in the present study (Fig. 1).

CUPRAC assay showed a range of 0.13 ± 0.001–0.65 ± 0.007 mmol Trolox g^−1^ for oil-bearing rose in this study. In general, the highest CUPRAC value was found in stage I (0.65 ± 0.007) and it decreased significantly to 0.13 ± 0.001 at stage V of flower development (Fig. 1a). There was no significant statistical difference between the last stages (stages IV and V) of flower development. The DPPH**·** scavenging activity decreased from 0.68 ± 0.002 to 0.55 ± 0.004 mmol Trolox g^−1^ throughout the flower development of oil-bearing rose. While DPPH**·** antioxidant capacity measurement results were statistically same for the first two stages, it decreased from 0.68 to 0.58 mmol Trolox g^−1^ from stage II to stage III and continued to decline from stage III to IV and did not change in stage V of flower development (Fig. 1b). The antioxidant capacity measured by FRAP assay showed a similar pattern to CUPRAC assay (Fig. 1c). FRAP values were between 0.22 ± 0.009 and 0.83 ± 0.05 mmol Trolox g^−1^. As shown in Fig. 1c, the FRAP value was 0.83 mmol Trolox g^−1^ at stage I, then decreased significantly until stage III (0.24 ± 0.01) and remained unchanged between stage III to V. The FIC scavenging activity of oil-bearing rose petals at different developmental stages was found to be ranging from 0.35 ± 0.01 to 0.42 ± 0.02 mmol EDTA g^−1^ (Fig. 1d). The highest FIC value was detected in stage I and II, and dropped significantly to 0.37 ± 0.001 at stage III and did not change the stages III to V. The highest ABTS activity among the five developmental stages was observed in Stage I (Fig. 1e) similar to the other methods. The ABTS values continuously decreased from 0.74 ± 0.04 to 0.16 ± 0.007 mmol Trolox g^−1^ from stage I to V. It reduced by 43% in stage II and 77% in stage III as compared to stage I.

### Antioxidant enzyme activities of oil-bearing rose petals at different stages of flower development

**Figure 2:**
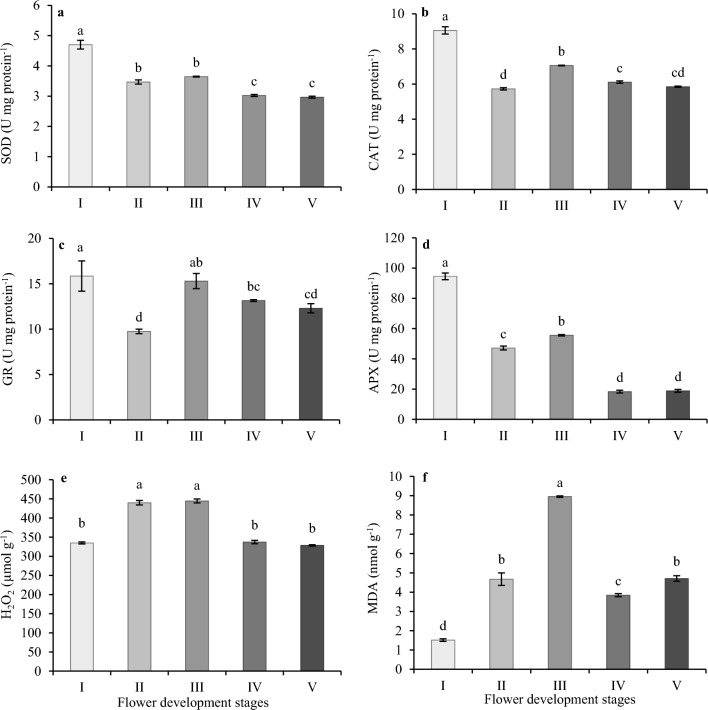
Antioxidant enzyme activities of oil-bearing rose petals during the flower development. (**a**) superoxide dismutase activity (SOD), (**b**) catalase (CAT), (**c**) glutathione reductase (GR), (**d**) Ascorbate peroxidase (APX), (**e**) hydrogen peroxide (H_2_O_2_), (**f**) malondialdehyde (MDA). Data are represented as mean ± standard deviation (n = 3). Mean values with a different letters were significantly different (p ≤ 0.05).

SOD activity decreased significantly from stages I to II and III, thereafter, the SOD activity decreased further at stage IV and remained the same at stage V (Fig. 2a). SOD activity was 35.5, 55.5, and 58.5% less at II, III and V stages, respectively, compared to stage I. The highest CAT activity was observed at stage I (9.06 U mg/protein) and III (7.05 U mg/protein), and the CAT activity was not significantly different at stages IV and V (Fig. 2b). The lowest CAT activity was observed at the stage II (5.73 ± 0.0.6 U mg protein^−1^) of flower development. GR activity showed a similar pattern to CAT activity during the flower development of oil-bearing rose, hence the GR activity was highest in stage I (15.84 ± 1.67 U mg protein^−1^) and was at the lowest in stage II (9.75 ± 0.24 U mg protein^−1^), and significantly increased at stage III (15.29 ± 0.834 U mg protein^−1^) (Fig. 2c). GR activity was not significantly different between the stage IV and V. APX activity also showed a similar activity pattern to SOD activity during flower development of oil-bearing rose plants (Fig. 2d). A significant decrease from stage I (94.53 ± 2.24) to stage II (47.15 ± 1.3) and a significant increase at stage III (55.6 ± 0.46) was observed. APX activity later dropped to 18.303 U mg protein^−1^ at stage IV and its activity did not change at stage V. The H_2_O_2_ content was significantly higher during the stage II (439.85 ± 5.9 μmol g^–1^) and stage III (444.54 ± 5.3 μmol g^–1^) compared to stage I. In contrast, a significant decrease in H_2_O_2_ (337.12 μmol g^–1^) was recorded in stage IV and stage V. However, no significant differences in H_2_O_2_ content were observed among the stages I, IV, and V (Fig. 2e). There was a regular increase in MDA content from stage I to III and the highest MDA content was detected at stage III (8.95 nmol g^–1^) compared to all other flower development stages. MDA content showed more than five-fold increase at stage III compared to stage I (Fig. 2f). While a significant decrease was detected from stage III to stage IV, on the contrary, a slight increase was observed from stage IV to stage V.

### Mineral composition of oil-bearing rose petals at different flowering stages

In order to determine the amount of mineral nutrients, we used high-throughput elemental analysis technology (ICP-OES), and found significant variations throughout the flower development stages (Fig. 5). Al and Se contents could only be quantitatively determined at stage II, and Hg, Cd, Cr, Cu and Fe were at such low levels that they could not be determined at any flower development stage (Table 1). Ca, K, Mg, Mn, P and Si had the highest accumulation at stage I and they had the lowest accumulation at stage V. Conversely, Zn was at the highest level at stage III and lowest at stage V. While the amount of Na was too low to be determined at stages I and III, Na was detected at stages II, IV and V, and the highest level of Na was observed at stage IV (0.042 mg g^−1^) of flower development. K was the most abundant mineral in all developmental stages, followed by P, Mg and Ca, respectively.

**Table 1:** Mineral composition (mg g^−1^ DW) of oil-bearing rose petals during the flower development determined by ICP-OES.

Elemental composition	Flower development stages				
	I	II	III	IV	V
Al	tr*	0.053 ± 0.02 a	tr	tr	tr
Ca	1.219 ± 0.02 a	0.752 ± 0.03 b	0.495 ± 0.04 c	0.573 ± 0.01 c	0.316 ± 0.06 d
Hg	tr	tr	tr	tr	tr
Cd	tr	tr	tr	tr	tr
Cr	tr	tr	tr	tr	tr
Cu	tr	tr	tr	tr	tr
Fe	tr	tr	tr	tr	tr
K	18.410 ± 0.19 a	15.790 ± 0.19 b	13.140 ± 0.23 c	15.840 ± 0.24 b	11.300 ± 1.52 c
Mg	1.978 ± 0.12 a	1.494 ± 0.04 b	1.209 ± 0.082 c	1.451 ± 0.04 b	0.729 ± 0.09 d
Mn	0.030 ± 0.01 a	0.021 ± 0.02 a	0.020 ± 0.001 a	0.019 ± 0.02 a	0.011 ± 0.001 a
Na	tr	0.019 ± 0.02 ab	tr	0.042 ± 0.005 a	0.009 ± 0.001 b
P	2.540 ± 0.02 a	2.240 ± 0.07 ab	1.586 ± 0.064 bc	1.730 ± 0.28 bc	1.184 ± 0.46 c
Se	tr	0.007 ± 0.001 a	tr	tr	tr
Si	0.030 ± 0.02 a	0.020 ± 0.02 a	0.015 ± 0.003 a	0.024 ± 0.02 a	tr
Zn	0.026 ± 0.002 ab	0.014 ± 0.001 ab	0.083 ± 0.06 a	0.017 ± 0.001 ab	0.008 ± 0.002 b

Data are represented as mean ± standard deviation (n = 3).

Mean separation within each row by least significant differences (LSD) at *P* ≤ 0.05. Means followed by the same letters are not significantly different from one another.

*tr = trace amount, less than < 0.005 mg g^−1^.

### The correlation analysis

**Figure 3:**
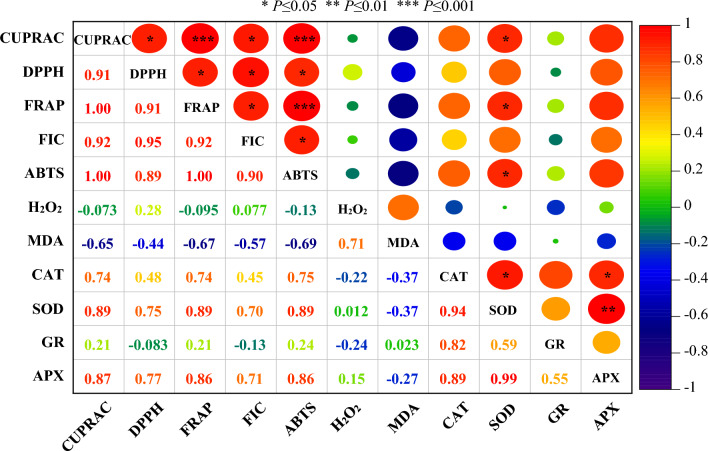
Correlations between antioxidant capacity and antioxidant enzyme activities generated by a heat map using mean values obtained from five flower development stages of oil-bearing rose. The color scale displays the intensity of the correlation coefficient values of the measured parameters. (*H*_*2*_*O*_*2*_ hydrogen peroxidase, *MDA* malondialdehyde, *CAT* catalase, *SOD* superoxide dismutase, *GR* glutathione reductase, *APX* ascorbate peroxidase).

Correlation analysis was carried out to determine the relationships between antioxidant enzyme activities and antioxidant capacity during flower development of oil-bearing rose. The result of correlation analysis for five different flower development stages of oil-bearing rose is shown in Fig. 3. Of the 66 coefficients, 16 were significant at the *P* ≤ 0.05, *P* ≤ 0.01, and *P* ≤ 0.001 levels. ABTS, FIC, FRAP, DPPH and CUPRAC, which are predictors of antioxidant capacity, were significantly and positively correlated with each other. APX was only positively correlated with CAT and SOD activity. SOD activity showed significant positive correlation with CUPRAC, FRAP, ABTS and CAT activity. MDA content was negatively correlated with antioxidant capacity, but it was positively correlated with H_2_O_2_ content.

**Figure 4:**
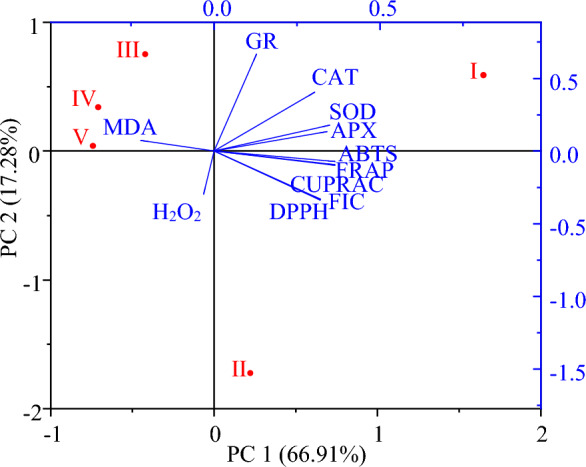
Principle component analysis (PCA) of antioxidant capacity and antioxidant enzyme activities during flower development of oil-bearing rose. (*H*_*2*_*O*_*2*_ hydrogen peroxidase, *MDA* malondialdehyde, *CAT* catalase, *SOD* superoxide dismutase, *GR* glutathione reductase, *APX* ascorbate peroxidase).

Changes in antioxidant capacity and antioxidant enzyme activities were further analyzed with PCA analysis during the flower development and the results were shown in Fig. 4. The two principal components (PC) represented 84.19% of the total variation (66.91% and 17.28% for PC1 and PC2, respectively). The flower development stages were divided into four clusters. PCA analysis showed that stages III, IV and V were closely related and associated with MDA accumulation. The effects of flower development stages on antioxidant capacity and enzymes were distributed along the PC1 axis in the order V > IV > III > II > I. GR, CAT, SOD and APX were significantly changed at stage I of flower development and clustered on the right side of the PC1 axis. Antioxidant capacity was significantly affected in stages I and II and clustered on the right side of the PC1 axis.

## Discussion

The antioxidant content and free radical scavenging properties of plants are used in the cosmetic industry to modulate skin damage^37^. Therefore, antioxidant-rich plants are widely used to protect against oxidative degradation. Recent decades have seen a rise in interest in nutrition and food science thanks to natural diets with antioxidant activity^38^. In general, the human health benefits of edible flowers are attributed to their antioxidant activity, namely their pronounced inhibitory effects on free radicals^39,40^. Since antioxidants in flowers are known as natural antioxidants, they are used as an alternative to prevent oxidative degradation of foods, thus minimizing the harm caused by these oxidative compounds in humans^41^. The important antioxidant activity in edible flower extracts, such as *Begonia, Tropaeolum* and *Rosa* were reported^42–44^.

Plants, due to differences in flower development, experience biochemical changes and ROS generation resulting in a decrease in antioxidant activity^45^. As flower development progressed in oil-bearing rose, SOD and CAT activity decreased, leading to O_2_^**·**–^ accumulation, resulting in increased MDA content at stage III. Contrary to our results, SOD activity increased in gladiolus (*Gladiolus grandiflora*) from early stages to stage IV of flower development, and then decreased after the blooming of flowers^17^. The activities of CAT and GR in oil-bearing rose showed a marginal decrease from the stage I to stage II, and showed a slight increase in stage III. Gladiolus flowers showed a continuous decline in CAT activity from bud stage through the full bloom stage^17^. In apples, CAT, SOD and peroxidase enzymes had higher activity during early stages of flower development^46^. It was possible that the increases in CAT activity at bud stage could have resulted from the accumulation of H_2_O_2_ due to a higher rate of respiration during the pre-flowering stage. While CAT, GR and APX activity was the highest at bud stage, H_2_O_2_ content was lower, which suggests that antioxidant enzyme activities were sufficiently high to scavenge excess H_2_O_2_ accumulation. Furthermore, radical scavenging activity showed an increased trend from bud to full flowering stages in safflower (*Carthamus tinctorius*) flowers^47^. The scavenging power of free radicals produced in the samples is correlated with the measured antioxidant capacity. The key enzyme, APX, is used by plants to detoxify H_2_O_2_ using the ascorbate glutathione cycle^48^. Shamsi et al.^49^ and Cheruth et al.^50^ reported that APX activity was significantly high at the pre-flowering stage and declined during the flowering and postflowering stages in date palm. The decrease in APX activity with development progression is due to decreased SOD activity^48^. Throughout flower development, the scavenging capacity decreased because of an increase in ROS accumulation and a decrease in the activity of antioxidant enzymes. The behavior of antioxidant enzymes at different stages of flower development in oil-bearing rose reflects their active role in managing during the developmental processes.

Loss of membrane permeability during blooming and developmental processes may be caused by the oxidation of already-existing membrane components, such as increased lipid peroxidation during flowering. Accordingly, the combined reduction of antioxidant capacity and free radical scavenging system as antioxidant enzyme activities during the open flowering stage may result in a higher accumulation of reactive oxygen species (ROS)^46,51^. However, the balance between H_2_O_2_ accumulation and neutralization changed hence antioxidant enzyme activities were statistically significantly reduced at stage II, and a significant increase in MDA level was also observed at stages II and III, which prompted an overall increase of antioxidant enzyme activities at stage III of flower development.

There are different methods to evaluate the antioxidant capacities in extracts, therefore five different methods were selected for the evaluation of the antioxidant activity in oil-bearing rose, which differs from each other in terms of substrates, probes, reaction conditions, and quantification methods. All five different methods showed that stage I of oil-bearing rose flowers showed the strongest antioxidant capacity, and flowers in stages IV and V had the lowest antioxidant capacity. Therefore, the best harvest time for oil-bearing rose flowers are stage I and II for the capture of the highest antioxidant capacity. Similar results have also been reported for apple cultivars (*Malus* spp.) and *R. hybridia* petals^52,53^. ABTS, DPPH and FRAP results revealed that apple petals at the bud stage exhibited much higher antioxidant activity compared to the other four developmental stages. However, the antioxidant capacity of daylily flowers (*Hemerocallis fulva Linn.)* showed an increasing trend during flower development^42^. Concerning reducing power, chelating power and lipoperoxidation inhibition capacities, borage flower (*Borago officinalis* L.) extracts showed an increase in antioxidant levels during flower development and reached their maximum at full bloom^54^. The strong antioxidant capacity of oil-bearing rose flowers at bud stages suggests that they can be used as a source of bioactive substances, and they could have great potential to improve human health. However, due to the fact that the DPPH radical is unstable and can only respond to lipophilic antioxidants^55^, it showed a different tendency to decrease compared to other antioxidant capacities during the development of oil-bearing rose. Therefore, it is recommended to perform two or more antioxidant capacity tests in studies.

In addition, since phenolic compounds are important plant components with redox properties responsible for antioxidant capacity, there is an important linear relationship between total phenolic components and antioxidant capacities^21,56^. In our previous study^57^, it was determined that the polyphenols content of the oil rose decreased during the development of the oil-bearing rose, and in the current study, the CUPRAC, FRAP and ABTS contents decreased during the development of the oil-bearing rose. When the results of these two studies are examined together, the hypothesis that there is a linear relationship between antioxidant capacity (CUPRAC, FRAP and ABTS) and polyphenol content in oil-bearing rose is supported.

Mineral concentrations in the petals of oil-bearing rose varied throughout by the developmental stages of flowers. The mineral composition shows that K was the most abundant mineral at all flower developmental stages, followed by P, Mg, Ca, Mn, Si and Zn. For the main effects of flowering, the maximum amount of Ca, K, Mg, Mn, P and Si concentrations were at bud stage, while the minimum was at fully opened flowers stage. Stage I of oil-bearing rose flower development had the highest concentrations for the mineral compounds, which have various potential health benefits as they affect homeostasis and metabolism^58^. Such high mineral content at the bud stage may be linked to a better accumulation of these nutrients in the early days of flower development and potentially a lower vegetative-generative growth rate. Ca plays an important role in many plant signal transduction pathways^59^, cell division and cell wall formation^60^. Therefore, higher Ca concentration may be associated with petal development and expansion from bud stage to full bloom in oil-bearing rose flowers. In Opuntia flowers, it was determined that the K content decreased at full flowering stage compared to the initial flowering, while Ca, Mg and Na contents increased^61^. As petals contain less than 0.005 mg g^−1^ of Cd and Cr, the levels are within acceptable limits when compared to other edible flowers^62^. These toxic elements in the petals at all stages of development showed concentrations below the maximum tolerable limit, which is not harmful to human health in terms of toxicity.

Multivariate analysis methods such as heat-mapped correlation and PCA can show the effects of developmental stages of oil-bearing rose flowers on antioxidant activity and antioxidant capacity. A strongly significant correlation (*r*^2^ = 0.94) was observed between the DPPH and FRAP tests in different Zhongyuan tree flowers, concluding that the two methods for Zhongyuan tree flowers were comparable^63^. A positive correlation (0.874 ≤ *r*^2^ ≤ 0.968) was found between the antioxidant capacities determined by FRAP, DPPH and ABTS methods during the development of loquat (*Eriobotrya japonica*) flowers^64^. Similarly, Wong et al.^65^ determined a strong correlation between DPPH and FRAP methods. Positive correlations were observed between the antioxidant capacity of the oil-bearing rose petals measured by various methods (CUPRAC, DPPH, FRAP and ABTS). However, a strong correlation was observed between FIC activity and all antioxidant capacities, which may indicate that antioxidants may be the main chelators of ferrous ions. So far, a clear relationship between antioxidant activity and ferrous ion chelating activities has not been reported. PCA revealed the accumulation of antioxidant capacity and antioxidant enzyme activities, as well as decreased capacity and activity of antioxidants from stage III of flower development.

## Conclusion

Antioxidant capacity, antioxidant enzyme activities and mineral compositions were associated with the flowering stages of oil-bearing rose. Our results demonstrated that the antioxidant capacity, as measured by CUPRAC, DPPH, FRAP, FIC and ABTS methods, was at the maximum level at bud stage and then significantly decreased throughout flower development. Our results also showed that the levels of antioxidant capacity are different due to different stages of flower development. The activities of SOD, CAT, GR and APX were the highest at stage I and the lowest at stage IV and V. Mineral composition of petals changed depending on the development stage in oil-bearing rose flowers. The antioxidants found in oil-bearing rose flowers at different stages of development have strong development potential for market applications in the nutraceutical or cosmetic industries. These results can provide a theoretical basis for further use of oil-bearing rose flower sources. The antioxidant abilities of oil-bearing rose flowers may have great potential in preventing diseases associated with free radicals. Further research on oil-bearing rose flowers is appropriate to determine whether it has any effect on oxidative stress-related diseases.

## Materials and methods

**Figure 5:**
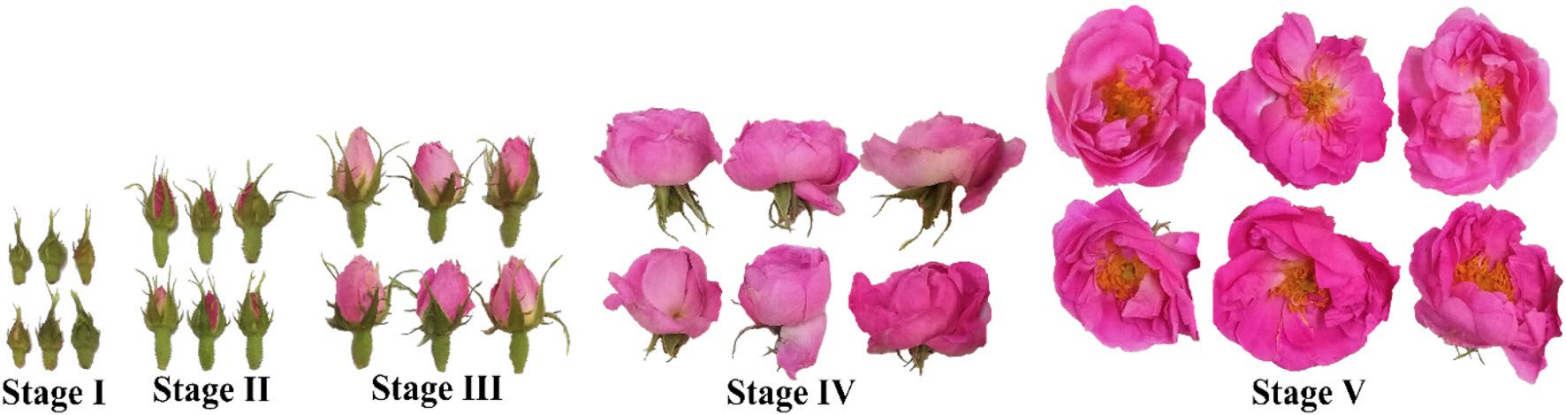
Five developmental stages of oil-bearing rose flowers used in the study.

Oil-bearing rose (*Rosa damascena* Mill. var. *trigintipetale*) flowers were hand-picked in a plantation grown for commercial purposes in Ardıçlı village (latitude 37° 48′ 19.9″ (N), longitude 30° 12′ 34.7″ (E), 906 m altitude) in Isparta, Türkiye. To minimize the effects of environmental conditions, rose flowers were collected in the early morning hours (06:00–08:00 AM). Five developmental stages were differentiated based on opening state of sepals and petals as described by Önder et al.^57^ (Fig. 5). All samples were collected in three replicates, and taken to the laboratory within an hour after the harvest, and stored at − 80 °C until analysis.

### Measuring the total antioxidant capacity of oil-bearing rose petals

For measuring antioxidant capacity with five different methods were used (CUPRAC, DPPH, FRAP, ABTS and FIC). Petals (1 g) were ground in liquid nitrogen, and homogenized in 10 mL of ice-cold 80% methanol. The homogenate was shaken on a rotary shaker for 10 min, then centrifuged at 15,000×*g* for 20 min at 4 °C. This procedure was repeated twice and the supernatants were pooled, brought to 25 mL volume with 80% methanol. The pooled extracts were stored at − 20 °C until the determination of the antioxidant capacity. All antioxidant capacity results were calculated on a fresh weight (FW) basis.

### Cupric reducing antioxidant capacity (CUPRAC) assay

The CUPRAC test was performed according to the method of Apak et al.^66^. CUPRAC reactions were set up as follows: 1 mL of 0.01 M copper (II) chloride, 1 mL of 0.0075 M neocuproine solution and 1.0 mL of 1 M ammonium acetate buffer solution (pH 7.0) were added successively into a glass tube. Subsequently, 0.2 mL of the 40-fold diluted extract solution was taken and 0.9 mL of deionized water was added to obtain a total volume of 4.1 mL and mixed well. Absorbance against a reagent solution without extract was measured at 450 nm (Shimadzu UV-1280, Kyoto, Japan) after 30 min. The antioxidant activity was calculated as Trolox equivalents per g of fresh weight (mmol TR g^−1^ FW) and calculated according to the following equation:

$$CUPRAC \left({\mathrm{mmol} \mathrm{TR} \mathrm{g}}^{-1}\right)=\frac{A}{{\varepsilon }_{TR}}\times \frac{{V}_{m}}{{V}_{s}}\times { D}_{f} \times \frac{{V}_{E}}{m}$$

where; *A*: sample absorbance measured at 450 nm; *Ɛ*_*TR*_: molar absorption coefficient of TR compound in the CUPRAC method (1.67 × 10^4^ L mol^−1^ cm^−1^); *Vm*: total volume (mL) of CUPRAC method measuring solution; *Vs*: sample volume (mL); *Df*: dilution factor (if needed); *V*_*E*_: volume of the prepared extract (mL); *m*: the amount of sample taken in the extraction process (g).

### 2,2-diphenyl-1-picrylhydrazyl (DPPH) assay

DPPH radical-scavenging activity was measured according to Bener et al.^67^. DPPH method was applied as follows: 1 mL of 40-fold diluted extract solution, 1 mL 99% ethanol and 2 mL of 0.2 mM of DPPH^**·**^ solution were added to a glass tube and mixed well. The reaction mixture was incubated at room temperature in the dark for 30 min. The absorbance of the samples was measured at 515 nm against ethanol with a UV–vis spectrophotometer (Shimadzu UV-1280, Kyoto, Japan). Free radical-scavenging activity was expressed as Trolox equivalents per g of fresh weight (mmol TR g^−1^ FW) and calculated according to the following equation:

$$DPPH \left({\mathrm{mmol} \mathrm{TR} \mathrm{g}}^{-1}\right)=\frac{{\Delta }_{A}}{{\varepsilon }_{TR}}\times \frac{{V}_{m}}{{V}_{s}} \times { D}_{f} \times \frac{{V}_{E}}{m}$$

where *Ɛ*_*TR*_: molar absorption coefficient of TR compound in the DPPH method (2.17 × 10^4^ L mol^−1^ cm^−1^); *V*_*s*_: sample volume (mL); *V*_*m*_: total volume (mL) of DPPH method measuring solution; *Df*: dilution factor (if needed); *V*_*E*_: volume of the prepared extract (mL); *m*: the amount of sample taken in the extraction process (g).

### Ferric reducing antioxidant power (FRAP) assay

The ferric reducing antioxidant power was measured according to the method of Berker et al.^68^. The following reactions were set up to measure FRAP activity: 1 mL of 50-fold diluted extract solution, 2.5 mL of 0.2 M phosphate buffer (pH 6.6) and 2.5 mL of 1% potassium ferricyanide solution were added to a glass tube and incubated for 20 min in a water bath at 50 °C. After incubation, 2.5 mL of 10% trichloroacetic acid (TCA) was added, and thoroughly mixed. An aliquot of 2.5 mL solution was mixed with 2.5 mL of distilled water and 0.5 mL of 0.1% iron (II) chloride solution; then the absorbance of the resulting Prussian blue solution was measured at 700 nm (Shimadzu UV-1280, Kyoto, Japan) after 2 min against a reagent blank. Ferric reducing antioxidant power activity was expressed as Trolox equivalents per g of fresh weight (mmol TR g^−1^ FW) and calculated according to the following equation:

$$FRAP \left({\mathrm{mmol} \mathrm{TR} \mathrm{g}}^{-1}\right)=\frac{A}{{\varepsilon }_{TR}}\times \frac{{V}_{m}}{{V}_{s}} \times { D}_{f} \times \frac{{V}_{E}}{m}$$

where; *A*: sample absorbance measured at 700 nm; *Ɛ*_*TR*_: molar absorption coefficient of TR compound in the FRAP method (1.77 × 10^4^ L mol^−1^ cm^−1^); *Vm*: total volume (mL) of FRAP method measuring solution; *Vs*: sample volume (mL); *Df*: dilution factor (if needed); *V*_*E*_: volume of the prepared extract (mL); *m*: the amount of sample taken in the extraction process (g).

### 2,2'-Azino-bis-(3-ethylbenzothiazoline-6-sulfonic acid) (ABTS) assay

The ABTS was measured according to the method of Re et al.^69^. ABTS^**·**+^ (7 mM) was dissolved in water and subsequently reacted with 2.45 mM potassium persulfate at a ratio of 1:0.5 to produce a radical cation. It was then incubated for 12–16 h in the dark at room temperature for the oxidation of ABTS. Before starting the analysis, ABTS^**·**+^ radical solution was diluted in ethanol until an absorbance of 0.7 ± 0.02 at 734 nm was obtained. ABTS method was applied as follows: 1 mL of 100-fold diluted extract solution, 3 mL deionized water and 1 mL of 7 mM of ABTS^**·**+^ solution were added to a glass tube and mixed well. Absorbance against a reagent solution without a sample was measured at 734 nm with a UV–Vis spectrophotometer (Shimadzu UV-1280, Kyoto, Japan) after 6 min. Trolox equivalents per g of fresh weight (mmol TR g^−1^ FW) and calculated according to the following equation:

$$ABTS \left({\mathrm{mmol} \mathrm{TR} \mathrm{g}}^{-1}\right)=\frac{\Delta A}{{\varepsilon }_{TR}}\times \frac{{V}_{m}}{{V}_{s}}\times { D}_{f} \times \frac{{V}_{E}}{m}$$

where *Ɛ*_*TR*_: molar absorption coefficient of TR compound in the ABTS method (2.6 × 10^4^ L mol^−1^ cm^−1^); *V*_*s*_: sample volume (mL); *V*_*m*_: total volume (mL) of ABTS method measuring solution; *Df*: dilution factor (if needed); *V*_*E*_: volume of the prepared extract (mL); *m* the amount of sample taken in the extraction process (g).

### Ferrous ion-chelating (FIC) assay

The ferrous ion-chelating ability was determined in accordance with a method described by Decker and Welch^70^ with minor modifications. FIC reactions contained 1 mL of extract solution, 3.7 mL of distilled water and 100 μL of 2 mM iron (II) chloride. The reaction was initiated by the addition of 200 μL of 5 mM ferrozine. The reaction was well mixed and incubated for 20 min at room temperature. After incubation, absorbance was determined at 562 nm (Shimadzu UV-1280, Kyoto, Japan) against a blank. Distilled water (1 mL) was used as a blank instead of the ferrozine solution, which helped with error correction due to the uneven color of the sample solutions. FIC was calculated with the following formulae:

$$FIC \left({\mathrm{mmol} \mathrm{EDTA} \mathrm{g}}^{-1}\right)=\frac{\Delta A}{{\varepsilon }_{EDTA}}\times \frac{{V}_{m}}{{V}_{s}} \times { D}_{f} \times \frac{{V}_{E}}{m}$$

where *Ɛ*_*EDTA*_: molar absorption coefficient of EDTA compound in the ABTS method (13.5 × 10^4^ L mol^−1^ cm^−1^); *V*_*s*_: sample volume (mL); *V*_*m*_: total volume (mL) of FIC method measuring solution; *Df*: dilution factor (if needed); *V*_*E*_: volume of the prepared extract (mL); *m* the amount of sample taken in the extraction process (g).

### Determination of antioxidant enzyme activities in oil-bearing rose petals

For SOD, CAT, GR and APX extraction, petals (10 g) were ground in liquid nitrogen and homogenized in 25 mL of 100 mM ice-cold 50 mM sodium phosphate (pH 6.4) containing 0.5 g of polyvinylpolypyrrolidone. The APX extract contained 1 mM ascorbic acid in addition to the other components of the extraction buffer. The homogenate was centrifuged at 15,000×*g* for 20 min at 4 °C. The resulting supernatants were filtered through Miracloth (Merck, Germany) and stored in the dark at  − 20 °C until antioxidant enzyme activities were determined in filtered aliquots.

SOD (EC 1.15.1.1) activity was measured by using the method of Giannopolitis and Ries^71^. The reaction mixture contained 2.8 mL reaction buffer (50 mM sodium phosphate buffer (pH 7.8), 13 mM methionine, 75 µM nitroblue tetrazolium (NBT), 0.1 mM EDTA, 50 mM sodium carbonate), 0.1 mL of 2 mM riboflavin, and 0.1 mL of the enzyme extract. The test tubes were irradiated under two fluorescent lamp (2 × 15 W) for 15 min, and absorbance was noted at 560 nm with a spectrophotometer (Shimadzu UV-1280, Kyoto, Japan). One unit of SOD activity was defined as the level of enzyme activity that caused a 50% decrease in SOD-inhibitable NBT reduction.

CAT (EC 1.11.1.6) activity was measured by following the method of Chance and Maehly^72^. The 3 mL reaction mixture contained 100 µL of enzyme extract and 2.9 ml of sodium phosphate buffer (50 mM, pH 7.0) containing 40 mM hydrogen peroxide (H_2_O_2_). Decreasing absorbance for H_2_O_2_ (Ɛ_240_ = 36 M^−1^ cm^−1^) was recorded at 240 nm (Shimadzu UV-1280, Kyoto, Japan) for 3 min. One unit of CAT activity was defined as H_2_O_2_ decomposition per minute.

GR (EC 1.6.4.2) activity was determined following the method of Murshed et al.^73^ with minor modifications. The 1 mL reaction mixture contained 100 µL of enzyme extract, 350 µL of sodium phosphate buffer (100 mM, pH 7.8) and 500 µL of 2 mM oxidized glutathione (GSSG). The reaction was initiated by adding 50 µL of 2.4 mM NADPH solution. Decreasing absorbance for NADPH (Ɛ_340_ = 6.22 mM^−1^ cm^−1^) was recorded at 340 nm (Shimadzu UV-1280, Kyoto, Japan) for 3 min. One unit of GR activity was defined as the amount of enzyme that caused the oxidation of 1 mmol NADPH per minute at pH 7.8 at 25 °C.

APX (EC 1.11.1.11) activity was measured by using the method of Nakano and Asada^74^. The 1 mL reaction mixture contained 100 µL of enzyme extract and 700 µL reaction buffer (sodium phosphate buffer (50 mM, pH 7.0), 0.5 mM ascorbate and 0.1 mM EDTA Na_2_). The reaction was initiated by adding 200 µL of 1.2 mM H_2_O_2_ solution. Decreasing absorbance for H_2_O_2_ (Ɛ_290_ = 2.8 mM^−1^ cm^−1^) was recorded at 290 nm (Shimadzu UV-1280, Kyoto, Japan) for 3 min. One unit of APX was defined as the amount of enzyme that caused the decomposition of 1 mmol H_2_O_2_ per minute at pH 7.0 at 25 °C.

All of the antioxidant enzyme activity was based on protein concentration; each activity result represents the average of three replications. The protein content was determined following Bradford method^75^. All specific antioxidant enzyme activities were expressed as U mg protein^−1^.

### Determination of H_2_O_2_ and malondialdehyde in oil-bearing rose petals

For H_2_O_2_ and malondialdehyde (MDA) extraction, petals (1 g) were ground in liquid nitrogen, homogenized in 10 mL of 0.1% trichloroacetic acid (TCA) and centrifuged at 13,000×*g* for 20 min at 4 °C. The resulting supernatants were used directly for the assays. H_2_O_2_ content was determined according to the method of Velikova et al.^76^. The reaction mixture contained 0.5 mL of 10 mM phosphate buffer (pH 7.0), 1 mL potassium iodide (1 M) and 0.5 mL of the extract. The reaction was developed for 1 h in dark. The absorption of the mixture was measured at 390 nm (Shimadzu UV-1280, Kyoto, Japan), and water was used as blank. A standard curve was used to determine the amount of H_2_O_2_ from known H_2_O_2_ concentrations. The H_2_O_2_ content was expressed as µmol g^−1^ FW.

MDA was determined according to the method of Heath and Packer^77^. The 1 mL of supernatant was added to 4.5 mL 0.5% 2-thiobarbituric acid (TBA) in 20% TCA. The mixture was boiled in a water bath for 30 min, and the reaction was stopped by cooling the reaction tubes on ice. Then the samples were centrifuged at 10,000×*g* for 10 min, the absorbance at 532 nm was measured and subtracted from the non-specific absorbance at 600 nm. The amount of MDA–TBA complex was calculated from the extinction coefficient 155 mM^−1^ cm^−1^.

### Determination of mineral compositions in oil-bearing rose petals

Mineral composition of petals were determined using Inductively coupled plasma-optical emission spectroscopy (ICP-OES). Samples were prepared by wet burning by adding 8 mL nitric acid + 2 mL H_2_O_2_ to 0.5 g of a sample using ETHOS ONE (Milestone, Italy) microwave sample preparation according to EPA 3015 method, and the final volume was completed to 20 mL with distilled water. Measurements in ICP-OES were performed using Optima 5300 DV Spectrometer (Perkin Elmer, USA) according to EPA 6010 method. In the petals, each element was measured at a specific wavelength (Al: 396.1, Ca: 317.9, Hg: 253.6, Cd: 228.8, Cr: 267.7, Cu: 327.4, Fe: 238.2, K: 766.5, Mg: 285.2, Mn: 257.6, Na: 589.6, P: 213.6, Se: 196.0, Si: 251.6 and Zn: 206.2 nm).

### Statistical analysis

All analyzes were performed with three replicates. Results were subjected to analysis of variance (ANOVA) using SPSS Statistics 22.0 software (IBM, Armonk, NY, USA). Least significant differences (LSD, *P* ≤ 0.05) were used to distinguish differences between the means. To show the relationship between the measured parameters, Pearson’s linear correlation analysis (Heatmap correlation) was calculated using OriginPro software (version 2021, OriginLab, Northampton, MA). The mean values of antioxidant capacity and antioxidant enzymes at different stages of flower development were used for multivariate principal component analysis (PCA). All values were expressed as mean ± standard deviation.

### Ethics declarations

The collection of plant material and the performance of experimental research on such plants complied with the national guidelines of (Türkiye).

## Data Availability

The datasets generated during and/or analysed during the current study are available from the corresponding author on reasonable request.
